# LncRNA-modulated autophagy in plaque cells: a new paradigm of gene regulation in atherosclerosis?

**DOI:** 10.18632/aging.103786

**Published:** 2020-11-04

**Authors:** Kun Ren, Xiao-Dan Xu, Xiao-Hai Yu, Meng-Qi Li, Meng-Wen Shi, Qi-Xian Liu, Ting Jiang, Xi-Long Zheng, Kai Yin, Guo-Jun Zhao

**Affiliations:** 1 The Sixth Affiliated Hospital of Guangzhou Medical University, Qingyuan City People’s Hospital, Qingyuan, Guangdong, China; 2 Department of Pathophysiology, School of Basic Medical Sciences, Anhui Medical University, Hefei, Anhui, China; 3 Department of Pathology, The First Affiliated Hospital of Anhui Medical University, Hefei, Anhui, China; 4 Department of Biochemistry and Molecular Biology, Libin Cardiovascular Institute of Alberta, University of Calgary, Health Sciences Center, Calgary, AB, Canada; 5 Key Laboratory of Molecular Targets and Clinical Pharmacology, School of Pharmaceutical Sciences, Guangzhou Medical University, Guangzhou, Guangdong, China; 6 The Second Affiliated Hospital of Guilin Medical University, Guangxi Key Laboratory of Diabetic Systems Medicine, Guilin Medical University, Guilin, China

**Keywords:** long noncoding RNAs, autophagy, atherosclerosis

## Abstract

The development of atherosclerosis is accompanied by the functional deterioration of plaque cells, which leads to the escalation of endothelial inflammation, abnormal vascular smooth muscle cell phenotype switching and the accumulation of lipid-laden macrophages within vascular walls. Autophagy, a highly conserved homeostatic mechanism, is critical for the delivery of cytoplasmic substrates to lysosomes for degradation. Moderate levels of autophagy prevent atherosclerosis by safeguarding plaque cells against apoptosis, preventing inflammation, and limiting the lipid burden, whereas excessive autophagy exacerbates cell damage and inflammation and thereby accelerates the formation of atherosclerotic plaques. Increasing lines of evidence suggest that long noncoding RNAs can be either beneficial or detrimental to atherosclerosis development by regulating the autophagy level. This review summarizes the research progress related to 1) the significant role of autophagy in atherosclerosis and 2) the effects of the lncRNA-mediated modulation of autophagy on the plaque cell fate, inflammation levels, proliferative capacity, and cholesterol metabolism and subsequently on atherogenesis.

## INTRODUCTION

Cardiovascular disease (CVD) is among the leading causes of morbidity and mortality around the world. The critical pathological foundation of CVD is atherosclerosis, which is characterized by the formation of atheromatous plaques in large- and medium-sized arteries, a damaged endothelium, fatty deposits and the build-up of fibrous caps [1, 2]. The development of strategies for the prevention of atherosclerosis will facilitate a decrease in the incidence of CVD.

Autophagy, an evolutionarily conserved catabolic process, is the orderly turnover of cellular components that deliver harmful pathogens, damaged protein aggregates and organelles to the lysosome for degradation [3]. Basal autophagy protects cells against the accumulation of cytosolic metabolic waste or other dysfunctional constituents. Under environmentally critical conditions, autophagy-mediated nutrient recycling is crucial for the maintenance of energy sources and cell viability [4]. Specifically, the entire process involves more than 30 types of autophagy-related genes (ATGs) and can generally be divided into the following three steps: (1) cytoplasmic materials are sequestered into an expanding membrane sac, called the phagophore, which matures into a characteristic double-membrane vesicle, known as the autophagosome; (2) autophagosomes fuse with lysosomes to form autolysosomes, where dysfunctional organelles and cellular substances are degraded; and (3) the degraded components are delivered to the cytoplasm for anabolism or recycling [5]. To date, three different forms of autophagy have been delineated: macroautophagy, chaperone-mediated autophagy, and microautophagy. In this review, macroautophagy is referred to as autophagy.

**Figure 1:**
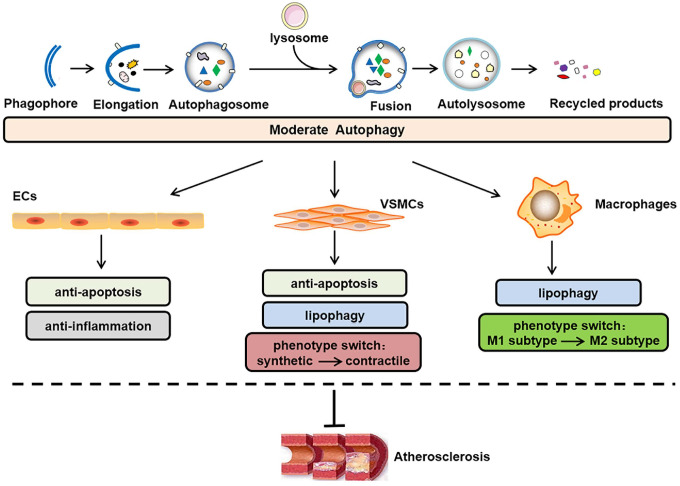
**Role of moderate autophagy in atherosclerosis.** The process of autophagy primarily involves the following steps: phagophore elongation, autophagosome formation, autophagosome-lysosome fusion, autolysosome formation, acidic hydrolase-mediated degradation of the autophagosome cargo and recycling of constituent macromolecules. In addition, moderate autophagy can inhibit atherosclerosis by protecting plaque cells (i.e., ECs, VSMCs, and macrophages) against apoptosis, inflammation, lipid accumulation, and abnormal phenotype switching. ECs, endothelial cells; VSMCs, vascular smooth muscle cells.

Autophagy occurs in all major atherosclerotic plaque cells present around the necrotic core and in the fibrous cap, e.g., endothelial cells (ECs), vascular smooth muscle cells (VSMCs) and macrophages [6]. In the normal vessel wall, basal/moderate levels of autophagy function as cytoprotective and antiatherogenic mechanisms [7], as illustrated in Figure 1. The sequestosome 1 (SQSTM1/p62) levels are considered as a negative indicator of autophagic flux [8, 9]. In Western diet-fed apoE-deficient (apoE^-/-^) mice, the SQSTM1/p62 levels are clearly amplified in the atherosclerotic aortae, and increases in the age/plaque burden are associated with further enhancements in these levels [10], which suggests that plaque progression is accompanied by defective autophagy. The conundrum of how reparative, life-sustaining machinery, such as autophagy, becomes dysfunctional in atherosclerosis has attracted the attention of researchers. In contrast to basal autophagy, the available evidence also shows that certain stimuli can induce excessive autophagy, which exerts cytotoxic effects on plaque cells and is detrimental for atherogenesis [11]. In general, the disruption of “autophagic homeostasis”, which can be reflected as either insufficient autophagy or overstimulated autophagy, is common during atherogenesis.

**Figure 2:**
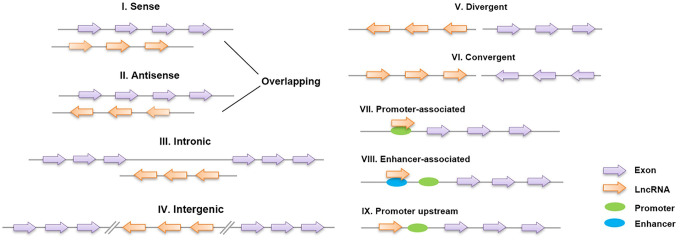
**Classification of lncRNAs based on their genomic region.** I, II: Sense lncRNAs and antisense lncRNAs are located on the same and opposite strands, respectively, and overlap with neighboring genes; III: intronic lncRNAs are transcribed entirely from the introns of protein-encoding genes; IV: intergenic lncRNAs lie within the genomic interval between two genes; V, VI: divergent/convergent lncRNAs are transcribed in the opposite/same direction as nearby protein-encoding genes; VII, VIII: promoter/enhancer-associated lncRNAs originate from the promoter/enhancer regions of protein-encoding genes; and IX: lncRNAs are situated upstream of the promoter.

**Figure 3:**
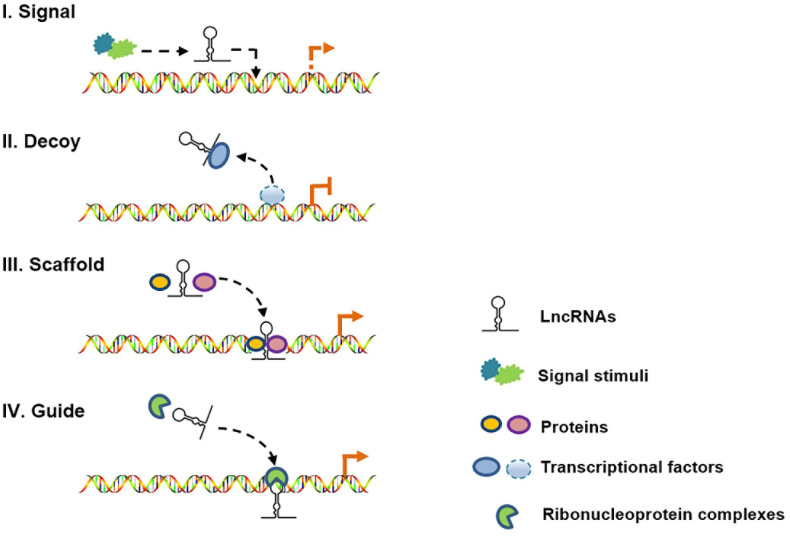
**Schematic diagram of the four mechanisms of action of lncRNAs.** (I) As molecular signals, lncRNAs are involved in gene transcription in response to various stimuli; (II) as decoys, lncRNAs can repress gene transcription by titrating transcription factors; (III) as scaffolds, lncRNAs can recruit different proteins to target genes; and (IV) as guides, lncRNAs can localize particular ribonucleoprotein complexes to specific chromatin targets.

Long noncoding RNAs (lncRNAs) are defined as non-protein-coding transcripts longer than 200 nucleotides [12]. According to their genomic location, lncRNAs can be classified into several broad subclasses, including sense/antisense, intronic, intergenic, divergent/convergent, promoter-/enhancer-associated and upstream promoter lncRNAs (Figure 2). In addition, lncRNAs can exhibit various functions, which include serving as molecular signals, decoys, guides and scaffolds (Figure 3) [13]. Despite their poor conservation among species, lncRNAs serve as molecular switches that determine the cell viability, inflammatory response and lipid metabolism in the vasculature by governing the autophagic flux [14, 15]. This review aims to provide a better overall picture of the relationships among lncRNAs, autophagy and atherosclerosis (Table 1).

**Table 1:** Take-home messages of this review.

**Non-coding RNAs**	**Cell type**	**Autophagy activity**	**As**
LncRNAs	Plaque cells (ECs, VSMCs, macrophages)	Basal/moderate level	↓
		Insufficient level	↑
		Excessive level	↑

**Notes:** LncRNAs can mitigate atherosclerosis development by inducing basal/moderate autophagy in plaque cells (ECs, VSMCs, macrophages) and instigate atherogenesis by inducing insufficient or excessive autophagy. EC, endothelial cell; VSMC, vascular smooth muscle cell; As, atherosclerosis.

↓, inhibitory effects; ↑, stimulatory effects.

## AUTOPHAGY IN ATHEROSCLEROSIS

### EC autophagy in atherogenesis

Based on the available evidence, the defective autophagy occurs in ECs during atherogenesis. ECs isolated from high-fat diet (HFD)-fed apoE^-/-^ mice exhibit significantly lower levels of ATG-6/Beclin-1 protein and a decreased ratio of microtubule-associated protein 1 light chain 3 II to I (LC3-II/I) [16]. In addition, low shear stress (LSS) can decrease the LC3-II/I ratio and the formation of autophagic vacuoles in human umbilical vein endothelial cells (HUVECs). The rapamycin-induced activation of autophagy promotes endothelial alignment in the flow direction, whereas the LSS-induced defects in autophagy disrupt this alignment and accelerate atherosclerotic plaque formation [17]. Nevertheless, Chen et al. reported that LC-3 and ATG-13 are highly expressed in aortic ECs of severe atherosclerotic patients, which indicates that the intimal ECs of advanced plaques might exhibit overactivated autophagy [18].

### Effects of autophagy on EC apoptosis

EC apoptosis contributes to atherogenesis [19]. Lunasin reportedly protects EAhy926 ECs against H_2_O_2_-induced cytotoxicity and mitochondrial-dependent apoptosis by repressing the generation of reactive oxygen species (ROS), which results in inhibition of the formation of thick plaques and increases in the collagen content in atherosclerotic lesions [20].

The role of endothelial autophagy in cell survival and atherosclerosis progression depends on the autophagy intensity levels. Wang et al. reported that the levels of ATG-5 and ATG-12 are decreased in ECs from the aortae of atherosclerotic mice. An *in vitro* study suggested that lower levels of ATG-5, ATG-12 and LC3-II proteins and enhanced SQSTM1/p62 accumulation are concomitant with an impaired cell viability of young HUVECs [21]. The injection of 6-amino-2,3-dihydro-3-hydroxymethyl-1,4-benzoxazine (ABO) into apoE^-/-^ mice substantially promotes autophagy and ameliorates apoptosis in the aortic endothelium. Furthermore, ABO robustly decreases the atherosclerotic plaque size and improves the stability of plaques [22]. Nevertheless, because autophagy is a process of self-cannibalization, it is reasonable to propose that under proatherosclerotic conditions, high levels of specific stimulators, such as oxidized low-density lipoproteins (ox-LDL) or lipopolysaccharides (LPS), might induce excessive “self-eating” and autophagic cell death [18, 23]. Peng et al. found that the treatment of HUVECs with 3BDO, a novel activator of mammalian target of rapamycin (m-TOR), can significantly reverse ox-LDL-induced autophagy. The administration of 3BDO to apoE^-/-^ mice decreases the ATG-13 protein level in the plaque endothelium, prevents EC death and stabilizes atherosclerotic lesions [24]. Similarly, recombinant thrombomodulin can exert antiatherogenic effects by inhibiting ox-LDL-induced EC apoptosis through the repression of overactivated autophagy [18].

### Effects of autophagy on EC-related inflammation

Pankratz et al. observed that miR-100 can suppress m-TOR complex 1 (mTORC1) signaling, enhance endothelial autophagy and decrease the expression of adhesion molecules *in vivo* and *in vitro*. In LDL receptor-deficient (LDLR^-/-^) mice, systemic miR-100 overexpression reduces the plaque area by 45% [25]. Additionally, apoE^-/-^ mice treated with morin hydrate exhibit attenuated atherosclerosis development, decreased serum levels of pro-inflammatory factors (TNF-α, ICAM-1) and enhanced autophagy in plaques. *In vitro* studies have revealed that morin hydrate-treated HUVECs display decreased inflammation, and these effects can be counteracted by the autophagy blocker 3-MA, which suggests that the anti-atherogenic and anti-inflammatory effects of morin hydrate are largely associated with the induction of autophagy [26].

### VSMC autophagy in atherogenesis

### Effects of autophagy on VSMC phenotype switching and proliferation

During atherosclerosis, VSMCs can switch from a differentiated, “contractile” phenotype to a dedifferentiated, “synthetic” phenotype, which is characterized by high proliferative and migratory capacities [27]. Injection of the DNA methyltransferase inhibitor (DNMT) 5-aza-2’-deoxycytidine into apoE^-/-^ mice can profoundly reduce the plaque size, and the underlying mechanisms are related to inhibition of the VSMC switch to a synthetic phenotype [28].

Autophagy suppresses the synthetic phenotype in VSMCs and cell proliferation [29]. Grootaert et al. showed that ATG-7 deficiency in VSMCs induces clear defects in autophagy and augments the proliferative capacity of the cells. In VSMC-specific ATG-7^-/-^ mice, the medial thickness of the aorta and the size of atherosclerotic plaques are significantly increased [30]. However, a positive correlation among autophagy, the synthetic VSMC phenotype and atherosclerosis has also been reported. The incubation of VSMCs with nicotine significantly promotes a switch from the contractile phenotype to the synthetic phenotype through the induction of autophagy. Injections of nicotine into HFD-fed apoE^-/-^ mice increase the LC3-II/I ratio, decrease the SQSTM1/p62 levels in plaque VSMCs and enlarge the lesion area in the aortic root [31]. The mechanisms underlying this contradiction merit further study.

Autophagy in VSMCs prevents cell death and attenuates the instability of atheromatous plaques [32]. VSMCs isolated from ATG-7^-/-^ mice exhibit a higher apoptotic death rate, and the mice had larger stenosis areas and intraplaque hemorrhage regions in their carotid arteries [33, 34]. Nevertheless, some factors can induce excessive VSMC autophagy and impair cell viability. The treatment of VSMCs isolated from human atherosclerotic plaques with TNF-α markedly enhances autophagic cell death by increasing the number of vacuolated cells and the expression of the autophagy marker LC3 [35]. In addition, human VSMCs treated with osteopontin exhibit marked increases in the autophagosome numbers, the LC3-II/I ratio and the expression of several ATGs, which results in accelerated cell death [36]. Altogether, the results show that moderate and overactivated autophagy most likely exert opposite effects on VSMC fate and atherosclerosis.

### Effects of autophagy on lipid accumulation in VSMCs

Autophagy can also impede atherogenesis by preventing excessive lipid loading in VSMCs. Li et al. observed that the downregulation of sterol regulatory element-binding cleavage-activating proteins (SREBPs) rescues dysfunctional autophagy in VSMCs, which leads to decreased accumulation of lipid droplets (LDs), free cholesterol (FC) and cholesteryl ester (CE). Furthermore, the VSMC-specific knockdown of SREBPs in apoE^-/-^ mice enhances cell autophagy and alleviates lipid deposition in plaques [37]. Similarly, the treatment of lipid-laden VSMCs with spermidine increases cholesterol efflux, and this effect is substantially attenuated in ATG-7-deficient VSMCs. *In vivo* studies have shown that spermidine significantly increases the LC3-II/I ratio, decreases the levels of SQSTM1/p62, and diminishes lipid deposition and necrotic core formation in aortic atherosclerotic plaques [38].

### Macrophage autophagy in atherogenesis

### Lipophagy

Lipophagy, a special type of autophagy, refers to an important acidic cholesterol hydrolysis pathway through which cytoplasmic LD-associated CE is transported to lysosomes [39]. This lipophilic flux consists of the following steps: (1) ATG-6/Beclin-1 mediates the formation of vesicle nucleation and subsequent LD-sequestered autophagosomes, which fuse with lysosomes to promote autolysosome production, and (2) lysosomal acid lipase (LAL) in the autolysosome hydrolyzes LD-associated CE to liberate FC [40]. Moreover, ATP-binding cassette transporter A1 (ABCA1) mediates the efflux of FC from macrophages, which is a key step in the formation of high-density lipoprotein (HDL) and reverse cholesterol transport (RCT) [40, 41]. RCT can increase the clearance of circulating low-density lipoprotein cholesterol (LDL-C), an independent risk factor for atherosclerosis [42, 43]. Thus, the activation of lipophagy is a viable therapeutic target for preventing lipid deposition and atherogenesis.

Jeong et al. observed dysfunctional lipophagy and an increased number of macrophage foam cells in plaques of peroxiredoxin-1^-/-^ apoE^-/-^ mice. In peroxiredoxin-1-deficient murine peritoneal macrophages, the ratio of autolysosomes to autophagosomes is decreased, which leads to impaired lipophagic flux and increased levels of total cholesterol (TC) and CE [44]. In addition, miR-33 antagonism exerts antiatherogenic effects by promoting the ABCA1-mediated efflux of cholesterol from plaque macrophages [45]. These effects are related to enhanced lipophagy, as demonstrated by the finding that hypercholesterolemic mice treated with miR-33 inhibitors show markedly increased LC3 levels, substantially higher autophagosome formation and less LD accumulation in macrophage foam cells [46]. These findings highlight the critical role of macrophage lipophagy in alleviating lipid overload and preventing atherogenesis.

### Effects of autophagy on macrophage polarization and inflammation

Liu et al. showed that ATG-5 deficiency suppresses anti-inflammatory M2 macrophage generation [47], which indicates that autophagy inhibits macrophage inflammation. Additionally, the cellular repressor of E1A-stimulated genes (CREG) can ameliorate macrophage inflammation by promoting lysosomal maturation and autolysosome formation. The treatment of HFD-fed apoE^-/-^ mice with recombinant CREG significantly decreases the plaque areas by reducing the number of macrophages and inhibiting inflammation in aortae [48]. Moreover, autophagy induced by tanshinone IIA promotes macrophage polarization towards the M2 phenotype. The administration of tanshinone IIA to apoE^-/-^ mice increases the CD206^+^ macrophage (M2) numbers, and this effect is accompanied by augmented autophagy, weakened inflammatory responses and smaller atherosclerotic lesion areas in the aorta [49].

## LNCRNAS INVOLVED IN ATHEROGENESIS THROUGH AUTOPHAGY

### EC

The downregulation of GAS5 prevents atherosclerosis progression in apoE^-/-^ mice [50]. In cardiac microvascular endothelial cells (CMECs), the overexpression of GAS5 significantly elevates the activity of the apoptosis-related protein caspase-3 and promotes the formation of incomplete nuclei [51]. Moreover, in ox-LDL-treated human aortic ECs, the knockdown of GAS5 substantially augments the LC3-II/I ratio, decreases the SQSTM1/p62 levels and reduces cell apoptosis, and these effects are reversed by miR-26a suppression. Therefore, impaired autophagic flux and exacerbated EC apoptosis might be involved in the pro-atherogenic effects of GAS5 [52]*.*

MALAT1 mitigates atherosclerotic plaque formation by decreasing myeloid cell adhesion to ECs and reducing proinflammatory cytokine production [53]. *In vitro* studies have shown that the downregulation of MALAT1 notably enhances the secretion of interleukin-6 (IL-6) and IL-8 from human aortic ECs [54]. The overexpression of MALAT1 significantly increases the LC3-II protein levels and facilitates the formation of autophagosomes and autolysosomes in HUVECs [55]. Similarly, the downregulation of MALAT1 in brain microvascular endothelial cells (BMECs) significantly decreases LC3-II expression and increases the SQSTM1/p62 levels by targeting the miR-200c-3p/sirtuin 1 (SIRT-1) axis [56]. Other studies have revealed that MALAT1 can also exert pro-autophagic effects through the miR-26b/ULK-2 axis [57] or the miR-216-5p/Beclin-1 axis [58]. MALAT1-induced autophagy in ECs might contribute to the inhibition of inflammation and atherogenesis.

Both LPS and ox-LDL can induce excessive autophagy and increase the TGFB2-OT1 levels [59]. TGFB2-OT1 can increase the production of IL-6 and IL-8 in HUVECs likely due to the overactivation of autophagy through the miR-4459/La ribonucleoprotein domain family member 1 pathway (LARP1) [59]. Whether TGFB2-OT1 accelerates atherogenesis by promoting autophagy and inflammation in ECs is unclear.

### VSMC

MALAT1 can inhibit atherosclerosis development through several mechanisms [60–62]. In siMALAT1-transfected VSMCs, the expression of SMC-specific contraction-related genes, including α-smooth muscle actin, SM-22, myocardin and serum response factor (SRF), was significantly enhanced. Furthermore, VSMC proliferation and migration are inhibited by the knockdown of MALAT1. Mechanistically, MALAT1 acts as a miR-142-3p sponge to enhance the expression of ATG-7 and LC3-II and decrease the SQSTM1/p62 levels [63]. Thus, in addition to its beneficial role in atherogenesis, MALAT1 might have the potential to accelerate atherosclerosis by inhibiting the contractile phenotype of VSMCs via autophagy stimulation.

The expression of the lncRNA BANCR is significantly increased in human atherosclerotic plaques. The overexpression of BANCR in VSMCs clearly increases the LC3-II/I ratio and promotes cell proliferation, and these effects are counteracted by the JNK inhibitor SP600125 [64]. Moreover, treatment with the autophagy inhibitor 3-MA significantly attenuates the positive effects of BANCR on autophagy and cell proliferation [65]. BANCR might exert pro-atherogenic effects by promoting VSMC autophagy and cell proliferation.

Guo et al. [66] found that apoE^-/-^ mice injected with lentiviral vector-siRNA-FA2H-2 exhibit defective autophagy, increased expression of mixed lineage kinase domain-like protein (MLKL), VCAM-1, MCP-1, and IL-6, and larger lesion areas in the aortic roots and aortic valves. *In vitro* studies have shown that the downregulation of FA2H-2 in VSMCs and ECs significantly increases the expression of MLKL, suppresses autophagic flux and amplifies the production of proinflammatory cytokines (IL-6, IL-8, IL-18, IL-1β, and TNF-α). Furthermore, pretreatment with 3-MA or ATG-7-shRNA reverses the effects induced by FA2H-2 knockdown, which demonstrates that FA2H-2 attenuates atherosclerotic plaque progression by enhancing autophagy in VSMCs and ECs to alleviate inflammation.

The lncRNA H19 reportedly exacerbates atherosclerosis and induces ischemic stroke [23]. The overexpression of H19 *in vivo* can increase VSMC inflammation [67]. Moreover, an inverse expression pattern of H19 and autophagy-related proteins (LC3-II/I ratio and Beclin-1) has been observed in both the thoracic aortae of apoE^-/-^ mice and human VSMCs. Further study showed that the H19-mediated suppression of the dual-specificity phosphatase 5 (DUSP-5)-ERK1/2 axis mitigates VSMC autophagy [68]. H19-induced atherogenesis is likely attributed to the repression of VSMC autophagy and the subsequent promotion of inflammation.

### Macrophage

The overexpression of DYNLRB2-2 in THP-1 macrophages markedly increases the ABCA1-mediated efflux of cholesterol and reduces intracellular LDs. The upregulation of DYNLRB2-2 also augments the expression of p-AMPK, LC3-II and ATG-6/Beclin-1 and decreases the levels of phospho-m-TOR and SQSTM1/p62. Furthermore, the effects of DYNLRB2-2 on macrophage lipid accumulation are attenuated by pretreatment with the AMPK signaling pathway inhibitor compound C or the autophagy blocker 3-MA [69]. Another study by Hu et al. revealed that DYNLRB2-2 enhances ABCA1-mediated lipid efflux and the anti-inflammatory response by amplifying the expression of G protein-coupled receptor 119 (GPR119) [70]. Further experiments are needed to verify whether DYNLRB2-2 protects against atherosclerosis through the enhancement of macrophage autophagy.

The TLR-4/NF-κB pathway has been well established as a facilitator of atherogenesis [71–73]. The available evidence shows that activation of the TLR-4/NF-κB pathway is positively correlated with the macrophage autophagy levels [74–77]. The lncRNA SNHG16 aggravates LPS-induced inflammation in mouse macrophages by upregulating TLR-4 expression [78]. The lncRNA HOTAIR promotes NF-κB activation and increases the expression of IL-6 and inducible nitric oxide synthase (iNOS) by accelerating IκBα degradation in RAW264.7 mouse macrophages [79]. The mechanism underlying the induction of inflammation by SNHG16 and HOTAIR might be related to overactivated autophagy. Additionally, MALAT1 overexpression inhibits the secretion of IFN-γ, TNF and IL-6 by increasing its interaction with the lncRNA NEAT1 in macrophages [62]. Another study performed by Zhao et al. [80] demonstrated that the MALAT1 levels in macrophages are upregulated by LPS, and MALAT1 reduces LPS-induced inflammation by acting as a negative feedback modulator of NF-κB. Because LPS is a potential inducer of excessive autophagy [59], MALAT1 might exert an anti-inflammatory effect in macrophages by repressing NF-κB-mediated autophagy.

## LNCRNAS INVOLVED IN THE DEVELOPMENT OF ATHEROSCLEROSIS-RELATED DISEASES THROUGH AUTOPHAGY

LncRNA-induced autophagy is also involved in other factors that influence the progression of arteriosclerosis, such as hypertension and obesity [81, 82]. Placental tissues from preeclampsia patients diagnosed with hypertension exhibit significantly higher H19 levels [83]. As observed in a mechanistic study, H19 overexpression decreases the viability of trophoblasts, increases the LC3-II/I ratio and the Beclin-1 levels and suppresses SQSTM1/p62 expression. H19 might aggravate hypertension by inducing excessive autophagy and cell death [84]. In addition, in human adipocyte-derived stem cells, the lncRNA MEG3 levels are decreased during adipocyte differentiation [85]. MEG3 overexpression significantly decreases adipocyte differentiation by targeting miR-140-5p [85]. The enhancement of autophagy facilitates adipogenic differentiation and increases the size of adipose depots [86–88], and miR-140-5p induces autophagy in different cell types [89, 90], which implies that MEG3 might alleviate obesity by inhibiting autophagy.

## LNCRNA-BASED THERAPEUTIC APPROACHES FOR CARDIOVASCULAR EVENTS

LncRNAs are appealing pharmacological targets due to their general cell type-specific functions, but many promising approaches for targeting lncRNAs remain in the preclinical phase [91]. Recombinant viral systems, such as adenoviruses and lentiviruses, are commonly used to deliver lncRNA transcripts into a target cell. The subcutaneous injection of the lncRNA CDKN2B-AS1-overexpressing lentiviral vector into apoE^-/-^ mice robustly mitigates atherosclerosis development by inhibiting ADAM10 [92]. The injection of adenovirus vectors containing lncRNA AZIN2-shRNA into the myocardium of rats significantly decreases the infarct size by sponging miR-214 and elevating PTEN expression [93]. Additionally, the intravenous injection of adenoviral-based siRNA against CHRF into an animal model antagonizes cardiac hypertrophy by targeting the miR-489/MyD-88 pathway [94]. Similarly, the intraperitoneal injection of lncRNA Chast-targeted GapmeR into mice significantly ameliorates cardiac remodeling and hypertrophy through the suppression of Plekhm1 expression [95]. Thus, the development of more therapeutic strategies targeting lncRNAs is critical for the treatment of CVD.

## CONCLUSIONS AND FUTURE DIRECTIONS

The entire process of atherogenesis is accompanied by autophagy impairment. Basal autophagy is beneficial in early atherosclerosis but severely dysfunctional in advanced atherosclerotic plaques. A beneficial level of autophagy in plaque cells, but not excessive autophagy, can protect cells against apoptosis, mitigate vascular inflammation and calcification, and alleviate lipid accumulation in atherosclerotic plaques. Additionally, autophagy can usually be overactivated in response to many stresses, including intracellular stresses (e.g., endoplasmic reticulum stress and reactive oxygen species) [96, 97], and extracellular stimuli (e.g., hypoxia, ox-LDL and LPS) [18, 23, 98]. In these cases, the cellular content is irreversibly depleted, which accelerates cell death and exacerbates the development of atherosclerosis [11, 32, 99]. LncRNAs participate in atherogenesis by acting as determinants of the autophagy status in plaque cells (i.e., ECs, VSMCs and macrophages) (Table 2). As described above, TGFB2-OT1 might induce overactivated autophagy in ECs and subsequently promote inflammation. SNHG16 and HOTAIR might induce atherogenesis by enhancing macrophage-induced inflammation through the overactivation of autophagy. The improper regulation of autophagy cannot reverse atherosclerotic plaque formation but rather worsens the atherosclerosis outcomes [100]. Thus, identifying the trend and pattern of autophagic responses during atherogenesis is of great significance for predicting the sensitivity/resistance of cells to pathological changes.

**Table 2:** LncRNAs that regulate autophagy in atherogenesis and the underlying mechanisms.

**Cell type**	**LncRNAs**	**Pathways**	**Autophagy**	**Cell function**	**As**	**Refs.**
EC	GAS5	miR-26a↓	↓	Apoptosis↑	↑?	50-52
	MALAT1	PI3K/AKT↓, miR-200c-3p↓/SIRT-1↑, miR-26b↓/ULK-2↑, miR-216-5p↓/Beclin-1↑	↑	Inflammation↓	↓?	53-58
	TGFB2-OT1	miR-4459↓/LARP1↑	↑↑?	Inflammation↑	↑?	59
	FA2H-2	MLKL↓	↑	Inflammation↓	↓	66
VSMC	MALAT1	miR-142-3p↓/ATG-7↑	↑	Synthetic phenotype↑	↑?	63
	BANCR	JNK↑	↑?	Proliferation and migration↑	↑?	64, 65
	FA2H-2	MLKL↓	↑	Inflammation↓	↓	66
	H19	DUSP5-ERK1/2↓	↓	Inflammation↑	↑?	67, 68
Macrophage	DYNLRB2-2	GPR119↑/ABCA1↑	↑	Foam cell formation↓, inflammation↓	↓?	69, 70
	SNHG16	TLR-4/NF-κB↑	↑↑?	Inflammation↑	↑?	78
	HOTAIR	NF-κB↑	↑↑?	Inflammation↑	↑?	79
	MALAT1	NEAT1↓, NF-κB↓	↓↓?	Inflammation↓	↓?	62, 80

**Notes:** ↑, stimulatory effects; ↑↑, excessive activation; ↓, inhibitory effects; ↓↓, restrain of excessive activation;?, needs to be confirmed.

**Abbreviations:** EC, endothelial cell; VSMC, vascular smooth muscle cell; As, atherosclerosis; SIRT-1, sirtuin 1; ULK-2, unc-51-like autophagy-activating kinase 2; LARP1, La ribonucleoprotein domain family member 1; MLKL, mixed lineage kinase domain-like protein; JNK, c-Jun N-terminal kinase; ATG-7, autophagy-related gene 7; DUSP-5, dual-specificity phosphatase 5; GPR119, G protein-coupled receptor 119; ABCA1, ATP-binding cassette transporter A1; TLR-4, Toll-like receptor 4; NF-κB, nuclear factor kappa-B.

The roles of other forms of autophagy in addition to macroautophagy, such as microautophagy, chaperone-mediated autophagy, mitophagy, aggrephagy, and xenophagy, in atherosclerosis have not been adequately addressed. By acting as critical determinants of autophagy, the lncRNA-miRNA and lncRNA-lncRNA axes exert profound influences on atherogenesis. Intriguingly, Liu et al. reported that the lncRNA NBR2 and AMPK kinase form a feed-forward loop under chronic energy stress conditions [101], which makes us wonder whether positive feedback loops between lncRNAs and autophagy components might promote the progression or remission of atherosclerosis. Unraveling these mechanisms will undoubtedly be helpful for the development of lncRNA-based drugs for atherosclerosis treatment.

## References

[1] Collura S, Morsiani C, Vacirca A, Fronterrè S, Ciavarella C, Vasuri F, D’Errico A, Franceschi C, Pasquinelli G, Gargiulo M, Capri M. The carotid plaque as paradigmatic case of site-specific acceleration of aging process: the microRNAs and the inflammaging contribution. Ageing Res Rev. 2020; 61:101090. 10.1016/j.arr.2020.101090 32474155

[2] Leong DP, Joseph PG, McKee M, Anand SS, Teo KK, Schwalm JD, Yusuf S. Reducing the global burden of cardiovascular disease, part 2: prevention and treatment of cardiovascular disease. Circ Res. 2017; 121:695–710. 10.1161/CIRCRESAHA.117.311849 28860319

[3] Mizushima N, Komatsu M. Autophagy: renovation of cells and tissues. Cell. 2011; 147:728–41. 10.1016/j.cell.2011.10.026 22078875

[4] Racanelli AC, Kikkers SA, Choi AM, Cloonan SM. Autophagy and inflammation in chronic respiratory disease. Autophagy. 2018; 14:221–32. 10.1080/15548627.2017.1389823 29130366 PMC5902194

[5] Yu L, Chen Y, Tooze SA. Autophagy pathway: cellular and molecular mechanisms. Autophagy. 2018; 14:207–15. 10.1080/15548627.2017.1378838 28933638 PMC5902171

[6] Liu H, Cao Y, Tong T, Shi J, Zhang Y, Yang Y, Liu C. Autophagy in atherosclerosis: a phenomenon found in human carotid atherosclerotic plaques. Chin Med J (Engl). 2015; 128:69–74. 10.4103/0366-6999.147815 25563316 PMC4837822

[7] De Meyer GR, Grootaert MO, Michiels CF, Kurdi A, Schrijvers DM, Martinet W. Autophagy in vascular disease. Circ Res. 2015; 116:468–79. 10.1161/CIRCRESAHA.116.303804 25634970

[8] Komatsu M, Waguri S, Koike M, Sou YS, Ueno T, Hara T, Mizushima N, Iwata J, Ezaki J, Murata S, Hamazaki J, Nishito Y, Iemura S, et al. Homeostatic levels of p62 control cytoplasmic inclusion body formation in autophagy-deficient mice. Cell. 2007; 131:1149–63. 10.1016/j.cell.2007.10.035 18083104

[9] Mathew R, Karp CM, Beaudoin B, Vuong N, Chen G, Chen HY, Bray K, Reddy A, Bhanot G, Gelinas C, Dipaola RS, Karantza-Wadsworth V, White E. Autophagy suppresses tumorigenesis through elimination of p62. Cell. 2009; 137:1062–75. 10.1016/j.cell.2009.03.048 19524509 PMC2802318

[10] Razani B, Feng C, Coleman T, Emanuel R, Wen H, Hwang S, Ting JP, Virgin HW, Kastan MB, Semenkovich CF. Autophagy links inflammasomes to atherosclerotic progression. Cell Metab. 2012; 15:534–44. 10.1016/j.cmet.2012.02.011 22440612 PMC3322320

[11] Levine B, Yuan J. Autophagy in cell death: an innocent convict? J Clin Invest. 2005; 115:2679–88. 10.1172/JCI26390 16200202 PMC1236698

[12] Nair L, Chung H, Basu U. Regulation of long non-coding RNAs and genome dynamics by the RNA surveillance machinery. Nat Rev Mol Cell Biol. 2020; 21:123–36. 10.1038/s41580-019-0209-0 32020081 PMC7107043

[13] St Laurent G, Wahlestedt C, Kapranov P. The landscape of long noncoding RNA classification. Trends Genet. 2015; 31:239–51. 10.1016/j.tig.2015.03.007 25869999 PMC4417002

[14] Zhao X, Su L, He X, Zhao B, Miao J. Long noncoding RNA CA7-4 promotes autophagy and apoptosis via sponging MIR877-3P and MIR5680 in high glucose-induced vascular endothelial cells. Autophagy. 2020; 16:70–85. 10.1080/15548627.2019.1598750 30957640 PMC6984615

[15] Li M, Cui J, Niu W, Huang J, Feng T, Sun B, Yao H. Long non-coding PCED1B-AS1 regulates macrophage apoptosis and autophagy by sponging miR-155 in active tuberculosis. Biochem Biophys Res Commun. 2019; 509:803–09. 10.1016/j.bbrc.2019.01.005 30621915

[16] Geng Z, Xu F, Zhang Y. MiR-129-5p-mediated beclin-1 suppression inhibits endothelial cell autophagy in atherosclerosis. Am J Transl Res. 2016; 8:1886–94. 27186312 PMC4859917

[17] Vion AC, Kheloufi M, Hammoutene A, Poisson J, Lasselin J, Devue C, Pic I, Dupont N, Busse J, Stark K, Lafaurie-Janvore J, Barakat AI, Loyer X, et al. Autophagy is required for endothelial cell alignment and atheroprotection under physiological blood flow. Proc Natl Acad Sci USA. 2017; 114:E8675–84. 10.1073/pnas.1702223114 28973855 PMC5642679

[18] Chen PS, Wang KC, Chao TH, Chung HC, Tseng SY, Luo CY, Shi GY, Wu HL, Li YH. Recombinant thrombomodulin exerts anti-autophagic action in endothelial cells and provides anti-atherosclerosis effect in apolipoprotein E deficient mice. Sci Rep. 2017; 7:3284. 10.1038/s41598-017-03443-z 28607460 PMC5468323

[19] Yang Q, Wang C, Jin Y, Ma X, Xie T, Wang J, Liu K, Sun H. Disocin prevents postmenopausal atherosclerosis in ovariectomized LDLR-/- mice through a PGC-1α/ERα pathway leading to promotion of autophagy and inhibition of oxidative stress, inflammation and apoptosis. Pharmacol Res. 2019; 148:104414. 10.1016/j.phrs.2019.104414 31449974

[20] Gu L, Ye P, Li H, Wang Y, Xu Y, Tian Q, Lei G, Zhao C, Gao Z, Zhao W, Tan S. Lunasin attenuates oxidant-induced endothelial injury and inhibits atherosclerotic plaque progression in ApoE^-/-^ mice by up-regulating heme oxygenase-1 via PI3K/Akt/Nrf2/ARE pathway. FASEB J. 2019; 33:4836–50. 10.1096/fj.201802251R 30601695

[21] Wang J, Wang WN, Xu SB, Wu H, Dai B, Jian DD, Yang M, Wu YT, Feng Q, Zhu JH, Zhang L, Zhang L. MicroRNA-214-3p: a link between autophagy and endothelial cell dysfunction in atherosclerosis. Acta Physiol (Oxf). 2018; 222:e12973. 10.1111/apha.12973 28888077

[22] Li H, Huang S, Wang S, Zhao J, Su L, Zhao B, Zhang Y, Zhang S, Miao J. Targeting annexin A7 by a small molecule suppressed the activity of phosphatidylcholine-specific phospholipase C in vascular endothelial cells and inhibited atherosclerosis in apolipoprotein E⁻/⁻mice. Cell Death Dis. 2013; 4:e806. 10.1038/cddis.2013.317 24052074 PMC3789175

[23] Huang Y, Wang L, Mao Y, Nan G. Long noncoding RNA-H19 contributes to atherosclerosis and induces ischemic stroke via the upregulation of acid phosphatase 5. Front Neurol. 2019; 10:32. 10.3389/fneur.2019.00032 30778327 PMC6369351

[24] Peng N, Meng N, Wang S, Zhao F, Zhao J, Su L, Zhang S, Zhang Y, Zhao B, Miao J. An activator of mTOR inhibits oxLDL-induced autophagy and apoptosis in vascular endothelial cells and restricts atherosclerosis in apolipoprotein E⁻/⁻mice. Sci Rep. 2014; 4:5519. 10.1038/srep05519 24980430 PMC4076681

[25] Pankratz F, Hohnloser C, Bemtgen X, Jaenich C, Kreuzaler S, Hoefer I, Pasterkamp G, Mastroianni J, Zeiser R, Smolka C, Schneider L, Martin J, Juschkat M, et al. MicroRNA-100 suppresses chronic vascular inflammation by stimulation of endothelial autophagy. Circ Res. 2018; 122:417–32. 10.1161/CIRCRESAHA.117.311428 29208678

[26] Zhou Y, Cao ZQ, Wang HY, Cheng YN, Yu LG, Zhang XK, Sun Y, Guo XL. The anti-inflammatory effects of morin hydrate in atherosclerosis is associated with autophagy induction through cAMP signaling. Mol Nutr Food Res. 2017; 61. 10.1002/mnfr.201600966 28421659

[27] Hénaut L, Mary A, Chillon JM, Kamel S, Massy ZA. The impact of uremic toxins on vascular smooth muscle cell function. Toxins (Basel). 2018; 10:218. 10.3390/toxins10060218 29844272 PMC6024314

[28] Zhuang J, Luan P, Li H, Wang K, Zhang P, Xu Y, Peng W. The yin-yang dynamics of DNA methylation is the key regulator for smooth muscle cell phenotype switch and vascular remodeling. Arterioscler Thromb Vasc Biol. 2017; 37:84–97. 10.1161/ATVBAHA.116.307923 27879253

[29] Grootaert MO, Moulis M, Roth L, Martinet W, Vindis C, Bennett MR, De Meyer GR. Vascular smooth muscle cell death, autophagy and senescence in atherosclerosis. Cardiovasc Res. 2018; 114:622–34. 10.1093/cvr/cvy007 29360955

[30] Grootaert MO, da Costa Martins PA, Bitsch N, Pintelon I, De Meyer GR, Martinet W, Schrijvers DM. Defective autophagy in vascular smooth muscle cells accelerates senescence and promotes neointima formation and atherogenesis. Autophagy. 2015; 11:2014–32. 10.1080/15548627.2015.1096485 26391655 PMC4824610

[31] Wang Z, Liu B, Zhu J, Wang D, Wang Y. Nicotine-mediated autophagy of vascular smooth muscle cell accelerates atherosclerosis via nAChRs/ROS/NF-κB signaling pathway. Atherosclerosis. 2019; 284:1–10. 10.1016/j.atherosclerosis.2019.02.008 30856513

[32] Tai S, Hu XQ, Peng DQ, Zhou SH, Zheng XL. The roles of autophagy in vascular smooth muscle cells. Int J Cardiol. 2016; 211:1–6. 10.1016/j.ijcard.2016.02.128 26954728

[33] Osonoi Y, Mita T, Azuma K, Nakajima K, Masuyama A, Goto H, Nishida Y, Miyatsuka T, Fujitani Y, Koike M, Mitsumata M, Watada H. Defective autophagy in vascular smooth muscle cells enhances cell death and atherosclerosis. Autophagy. 2018; 14:1991–2006. 10.1080/15548627.2018.1501132 30025494 PMC6152523

[34] Masuyama A, Mita T, Azuma K, Osonoi Y, Nakajima K, Goto H, Nishida Y, Miyatsuka T, Mitsumata M, Watada H. Defective autophagy in vascular smooth muscle cells enhances atherosclerotic plaque instability. Biochem Biophys Res Commun. 2018; 505:1141–47. 10.1016/j.bbrc.2018.09.192 30318118

[35] Jia G, Cheng G, Gangahar DM, Agrawal DK. Insulin-like growth factor-1 and TNF-alpha regulate autophagy through c-jun n-terminal kinase and Akt pathways in human atherosclerotic vascular smooth cells. Immunol Cell Biol. 2006; 84:448–54. 10.1111/j.1440-1711.2006.01454.x 16942488

[36] Zheng YH, Tian C, Meng Y, Qin YW, Du YH, Du J, Li HH. Osteopontin stimulates autophagy via integrin/CD44 and p38 MAPK signaling pathways in vascular smooth muscle cells. J Cell Physiol. 2012; 227:127–35. 10.1002/jcp.22709 21374592

[37] Li D, Chen A, Lan T, Zou Y, Zhao L, Yang P, Qu H, Wei L, Varghese Z, Moorhead JF, Chen Y, Ruan XZ. SCAP knockdown in vascular smooth muscle cells alleviates atherosclerosis plaque formation via up-regulating autophagy in ApoE^-/-^mice. FASEB J. 2019; 33:3437–50. 10.1096/fj.201800975RRR 30462530

[38] Michiels CF, Kurdi A, Timmermans JP, De Meyer GR, Martinet W. Spermidine reduces lipid accumulation and necrotic core formation in atherosclerotic plaques via induction of autophagy. Atherosclerosis. 2016; 251:319–27. 10.1016/j.atherosclerosis.2016.07.899 27450786

[39] Weidberg H, Shvets E, Elazar Z. Lipophagy: selective catabolism designed for lipids. Dev Cell. 2009; 16:628–30. 10.1016/j.devcel.2009.05.001 19460339

[40] Ouimet M, Franklin V, Mak E, Liao X, Tabas I, Marcel YL. Autophagy regulates cholesterol efflux from macrophage foam cells via lysosomal acid lipase. Cell Metab. 2011; 13:655–67. 10.1016/j.cmet.2011.03.023 21641547 PMC3257518

[41] Mo ZC, Ren K, Liu X, Tang ZL, Yi GH. A high-density lipoprotein-mediated drug delivery system. Adv Drug Deliv Rev. 2016; 106:132–47. 10.1016/j.addr.2016.04.030 27208399

[42] Gragnano F, Calabrò P. Role of dual lipid-lowering therapy in coronary atherosclerosis regression: evidence from recent studies. Atherosclerosis. 2018; 269:219–28. 10.1016/j.atherosclerosis.2018.01.012 29407597

[43] Klerkx AH, El Harchaoui K, van der Steeg WA, Boekholdt SM, Stroes ES, Kastelein JJ, Kuivenhoven JA. Cholesteryl ester transfer protein (CETP) inhibition beyond raising high-density lipoprotein cholesterol levels: pathways by which modulation of CETP activity may alter atherogenesis. Arterioscler Thromb Vasc Biol. 2006; 26:706–15. 10.1161/01.ATV.0000205595.19612.c9 16439711

[44] Jeong SJ, Kim S, Park JG, Jung IH, Lee MN, Jeon S, Kweon HY, Yu DY, Lee SH, Jang Y, Kang SW, Han KH, Miller YI, et al. Prdx1 (peroxiredoxin 1) deficiency reduces cholesterol efflux via impaired macrophage lipophagic flux. Autophagy. 2018; 14:120–33. 10.1080/15548627.2017.1327942 28605287 PMC5846566

[45] Rayner KJ, Sheedy FJ, Esau CC, Hussain FN, Temel RE, Parathath S, van Gils JM, Rayner AJ, Chang AN, Suarez Y, Fernandez-Hernando C, Fisher EA, Moore KJ. Antagonism of miR-33 in mice promotes reverse cholesterol transport and regression of atherosclerosis. J Clin Invest. 2011; 121:2921–31. 10.1172/JCI57275 21646721 PMC3223840

[46] Ouimet M, Ediriweera H, Afonso MS, Ramkhelawon B, Singaravelu R, Liao X, Bandler RC, Rahman K, Fisher EA, Rayner KJ, Pezacki JP, Tabas I, Moore KJ. microRNA-33 regulates macrophage autophagy in atherosclerosis. Arterioscler Thromb Vasc Biol. 2017; 37:1058–67. 10.1161/ATVBAHA.116.308916 28428217 PMC5494696

[47] Liu K, Zhao E, Ilyas G, Lalazar G, Lin Y, Haseeb M, Tanaka KE, Czaja MJ. Impaired macrophage autophagy increases the immune response in obese mice by promoting proinflammatory macrophage polarization. Autophagy. 2015; 11:271–84. 10.1080/15548627.2015.1009787 25650776 PMC4502775

[48] Sun M, Tian X, Liu Y, Zhu N, Li Y, Yang G, Peng C, Yan C, Han Y. Cellular repressor of E1A-stimulated genes inhibits inflammation to decrease atherosclerosis in ApoE(-/-) mice. J Mol Cell Cardiol. 2015; 86:32–41. 10.1016/j.yjmcc.2015.07.001 26163874

[49] Chen W, Li X, Guo S, Song N, Wang J, Jia L, Zhu A. Tanshinone IIA harmonizes the crosstalk of autophagy and polarization in macrophages via miR-375/KLF4 pathway to attenuate atherosclerosis. Int Immunopharmacol. 2019; 70:486–97. 10.1016/j.intimp.2019.02.054 30870679

[50] Meng XD, Yao HH, Wang LM, Yu M, Shi S, Yuan ZX, Liu J. Knockdown of GAS5 inhibits atherosclerosis progression via reducing EZH2-mediated ABCA1 transcription in ApoE^-/-^ mice. Mol Ther Nucleic Acids. 2020; 19:84–96. 10.1016/j.omtn.2019.10.034 31830648 PMC6926212

[51] Diao L, Bai L, Jiang X, Li J, Zhang Q. Long-chain noncoding RNA GAS5 mediates oxidative stress in cardiac microvascular endothelial cells injury. J Cell Physiol. 2019; 234:17649–62. 10.1002/jcp.28388 30825202

[52] Liang W, Fan T, Liu L, Zhang L. Knockdown of growth-arrest specific transcript 5 restores oxidized low-density lipoprotein-induced impaired autophagy flux via upregulating miR-26a in human endothelial cells. Eur J Pharmacol. 2019; 843:154–61. 10.1016/j.ejphar.2018.11.005 30468731

[53] Cremer S, Michalik KM, Fischer A, Pfisterer L, Jaé N, Winter C, Boon RA, Muhly-Reinholz M, John D, Uchida S, Weber C, Poller W, Günther S, et al. Hematopoietic deficiency of the long noncoding RNA MALAT1 promotes atherosclerosis and plaque inflammation. Circulation. 2019; 139:1320–34. 10.1161/CIRCULATIONAHA.117.029015 30586743

[54] Li S, Sun Y, Zhong L, Xiao Z, Yang M, Chen M, Wang C, Xie X, Chen X. The suppression of ox-LDL-induced inflammatory cytokine release and apoptosis of HCAECs by long non-coding RNA-MALAT1 via regulating microRNA-155/SOCS1 pathway. Nutr Metab Cardiovasc Dis. 2018; 28:1175–87. 10.1016/j.numecd.2018.06.017 30314869

[55] Li S, Pan X, Yang S, Ma A, Yin S, Dong Y, Pei H, Bi X, Li W. LncRNA MALAT1 promotes oxidized low-density lipoprotein-induced autophagy in HUVECs by inhibiting the PI3K/AKT pathway. J Cell Biochem. 2019; 120:4092–101. 10.1002/jcb.27694 30485490

[56] Wang S, Han X, Mao Z, Xin Y, Maharjan S, Zhang B. MALAT1 lncRNA induces autophagy and protects brain microvascular endothelial cells against oxygen-glucose deprivation by binding to miR-200c-3p and upregulating SIRT1 expression. Neuroscience. 2019; 397:116–26. 10.1016/j.neuroscience.2018.11.024 30496821

[57] Li Z, Li J, Tang N. Long noncoding RNA Malat1 is a potent autophagy inducer protecting brain microvascular endothelial cells against oxygen-glucose deprivation/reoxygenation-induced injury by sponging miR-26b and upregulating ULK2 expression. Neuroscience. 2017; 354:1–10. 10.1016/j.neuroscience.2017.04.017 28433650

[58] Wang K, Yang C, Shi J, Gao T. ox-LDL-induced lncRNA MALAT1 promotes autophagy in human umbilical vein endothelial cells by sponging miR-216a-5p and regulating beclin-1 expression. Eur J Pharmacol. 2019; 858:172338. 10.1016/j.ejphar.2019.04.019 31029709

[59] Huang S, Lu W, Ge D, Meng N, Li Y, Su L, Zhang S, Zhang Y, Zhao B, Miao J. A new microRNA signal pathway regulated by long noncoding RNA TGFB2-OT1 in autophagy and inflammation of vascular endothelial cells. Autophagy. 2015; 11:2172–83. 10.1080/15548627.2015.1106663 26565952 PMC4835209

[60] Li H, Zhu X, Hu L, Li Q, Ma J, Yan J. Loss of exosomal MALAT1 from ox-LDL-treated vascular endothelial cells induces maturation of dendritic cells in atherosclerosis development. Cell Cycle. 2019; 18:2255–2267. 10.1080/15384101.2019.1642068 31305205 PMC6738524

[61] Gao H, Wang X, Lin C, An Z, Yu J, Cao H, Fan Y, Liang X. Exosomal MALAT1 derived from ox-LDL-treated endothelial cells induce neutrophil extracellular traps to aggravate atherosclerosis. Biol Chem. 2020; 401:367–76. 10.1515/hsz-2019-0219 31318684

[62] Gast M, Rauch BH, Nakagawa S, Haghikia A, Jasina A, Haas J, Nath N, Jensen L, Stroux A, Böhm A, Friebel J, Rauch U, Skurk C, et al. Immune system-mediated atherosclerosis caused by deficiency of long non-coding RNA MALAT1 in ApoE-/-mice. Cardiovasc Res. 2019; 115:302–14. 10.1093/cvr/cvy202 30101304

[63] Song TF, Huang LW, Yuan Y, Wang HQ, He HP, Ma WJ, Huo LH, Zhou H, Wang N, Zhang TC. LncRNA MALAT1 regulates smooth muscle cell phenotype switch via activation of autophagy. Oncotarget. 2018; 9:4411–26. 10.18632/oncotarget.23230 29435112 PMC5796983

[64] Li H, Liu X, Zhang L, Li X. LncRNA BANCR facilitates vascular smooth muscle cell proliferation and migration through JNK pathway. Oncotarget. 2017; 8:114568–75. 10.18632/oncotarget.21603 29383102 PMC5777714

[65] Wang Y, Guo Q, Zhao Y, Chen J, Wang S, Hu J, Sun Y. BRAF-activated long non-coding RNA contributes to cell proliferation and activates autophagy in papillary thyroid carcinoma. Oncol Lett. 2014; 8:1947–52. 10.3892/ol.2014.2487 25289082 PMC4186573

[66] Guo FX, Wu Q, Li P, Zheng L, Ye S, Dai XY, Kang CM, Lu JB, Xu BM, Xu YJ, Xiao L, Lu ZF, Bai HL, et al. The role of the LncRNA-FA2H-2-MLKL pathway in atherosclerosis by regulation of autophagy flux and inflammation through mTOR-dependent signaling. Cell Death Differ. 2019; 26:1670–87. 10.1038/s41418-018-0235-z 30683918 PMC6748100

[67] Zhao Z, Sun W, Guo Z, Zhang J, Yu H, Liu B. Mechanisms of lncRNA/microRNA interactions in angiogenesis. Life Sci. 2020; 254:116900. 10.1016/j.lfs.2019.116900 31786194

[68] Song Z, Wei D, Chen Y, Chen L, Bian Y, Shen Y, Chen J, Pan Y. Association of astragaloside IV-inhibited autophagy and mineralization in vascular smooth muscle cells with lncRNA H19 and DUSP5-mediated ERK signaling. Toxicol Appl Pharmacol. 2019; 364:45–54. 10.1016/j.taap.2018.12.002 30529164

[69] Li Y, Sun T, Shen S, Wang L, Yan J. LncRNA DYNLRB2-2 inhibits THP-1 macrophage foam cell formation by enhancing autophagy. Biol Chem. 2019; 400:1047–57. 10.1515/hsz-2018-0461 30903747

[70] Hu YW, Yang JY, Ma X, Chen ZP, Hu YR, Zhao JY, Li SF, Qiu YR, Lu JB, Wang YC, Gao JJ, Sha YH, Zheng L, Wang Q. A lincRNA-DYNLRB2-2/GPR119/GLP-1R/ABCA1-dependent signal transduction pathway is essential for the regulation of cholesterol homeostasis. J Lipid Res. 2014; 55:681–97. 10.1194/jlr.M044669 24493833 PMC3966702

[71] Lu Z, Zhang X, Li Y, Lopes-Virella MF, Huang Y. TLR4 antagonist attenuates atherogenesis in LDL receptor-deficient mice with diet-induced type 2 diabetes. Immunobiology. 2015; 220:1246–54. 10.1016/j.imbio.2015.06.016 26162692 PMC4558266

[72] Ren K, Jiang T, Zhou HF, Liang Y, Zhao GJ. Apigenin retards atherogenesis by promoting ABCA1-mediated cholesterol efflux and suppressing inflammation. Cell Physiol Biochem. 2018; 47:2170–84. 10.1159/000491528 29975943

[73] Brown JD, Lin CY, Duan Q, Griffin G, Federation A, Paranal RM, Bair S, Newton G, Lichtman A, Kung A, Yang T, Wang H, Luscinskas FW, et al. NF-κB directs dynamic super enhancer formation in inflammation and atherogenesis. Mol Cell. 2014; 56:219–31. 10.1016/j.molcel.2014.08.024 25263595 PMC4224636

[74] Chuang SY, Yang CH, Chou CC, Chiang YP, Chuang TH, Hsu LC. TLR-induced PAI-2 expression suppresses IL-1β processing via increasing autophagy and NLRP3 degradation. Proc Natl Acad Sci USA. 2013; 110:16079–84. 10.1073/pnas.1306556110 24043792 PMC3791747

[75] Delgado MA, Elmaoued RA, Davis AS, Kyei G, Deretic V. Toll-like receptors control autophagy. EMBO J. 2008; 27:1110–21. 10.1038/emboj.2008.31 18337753 PMC2323261

[76] van der Vaart M, Korbee CJ, Lamers GE, Tengeler AC, Hosseini R, Haks MC, Ottenhoff TH, Spaink HP, Meijer AH. The DNA damage-regulated autophagy modulator DRAM1 links mycobacterial recognition via TLR-MYD88 to autophagic defense [corrected]. Cell Host Microbe. 2014; 15:753–67. 10.1016/j.chom.2014.05.005 24922577

[77] Sanjuan MA, Dillon CP, Tait SW, Moshiach S, Dorsey F, Connell S, Komatsu M, Tanaka K, Cleveland JL, Withoff S, Green DR. Toll-like receptor signalling in macrophages links the autophagy pathway to phagocytosis. Nature. 2007; 450:1253–57. 10.1038/nature06421 18097414

[78] Wang W, Lou C, Gao J, Zhang X, Du Y. LncRNA SNHG16 reverses the effects of miR-15a/16 on LPS-induced inflammatory pathway. Biomed Pharmacother. 2018; 106:1661–67. 10.1016/j.biopha.2018.07.105 30119242

[79] Obaid M, Udden SM, Deb P, Shihabeddin N, Zaki MH, Mandal SS. LncRNA HOTAIR regulates lipopolysaccharide-induced cytokine expression and inflammatory response in macrophages. Sci Rep. 2018; 8:15670. 10.1038/s41598-018-33722-2 30353135 PMC6199307

[80] Zhao G, Su Z, Song D, Mao Y, Mao X. The long noncoding RNA MALAT1 regulates the lipopolysaccharide-induced inflammatory response through its interaction with NF-κB. FEBS Lett. 2016; 590:2884–95. 10.1002/1873-3468.12315 27434861

[81] Tall AR, Yvan-Charvet L. Cholesterol, inflammation and innate immunity. Nat Rev Immunol. 2015; 15:104–16. 10.1038/nri3793 25614320 PMC4669071

[82] Bartolomaeus H, Balogh A, Yakoub M, Homann S, Markó L, Höges S, Tsvetkov D, Krannich A, Wundersitz S, Avery EG, Haase N, Kräker K, Hering L, et al. Short-chain fatty acid propionate protects from hypertensive cardiovascular damage. Circulation. 2019; 139:1407–21. 10.1161/CIRCULATIONAHA.118.036652 30586752 PMC6416008

[83] Xu J, Xia Y, Zhang H, Guo H, Feng K, Zhang C. Overexpression of long non-coding RNA H19 promotes invasion and autophagy via the PI3K/AKT/mTOR pathways in trophoblast cells. Biomed Pharmacother. 2018; 101:691–97. 10.1016/j.biopha.2018.02.134 29522949

[84] Shen F, Wei J, Snowise S, DeSousa J, Stone P, Viall C, Chen Q, Chamley L. Trophoblast debris extruded from preeclamptic placentae activates endothelial cells: a mechanism by which the placenta communicates with the maternal endothelium. Placenta. 2014; 35:839–47. 10.1016/j.placenta.2014.07.009 25096950

[85] Li Z, Jin C, Chen S, Zheng Y, Huang Y, Jia L, Ge W, Zhou Y. Long non-coding RNA MEG3 inhibits adipogenesis and promotes osteogenesis of human adipose-derived mesenchymal stem cells via miR-140-5p. Mol Cell Biochem. 2017; 433:51–60. 10.1007/s11010-017-3015-z 28382492

[86] Singh R, Xiang Y, Wang Y, Baikati K, Cuervo AM, Luu YK, Tang Y, Pessin JE, Schwartz GJ, Czaja MJ. Autophagy regulates adipose mass and differentiation in mice. J Clin Invest. 2009; 119:3329–39. 10.1172/JCI39228 19855132 PMC2769174

[87] Baerga R, Zhang Y, Chen PH, Goldman S, Jin S. Targeted deletion of autophagy-related 5 (atg5) impairs adipogenesis in a cellular model and in mice. Autophagy. 2009; 5:1118–30. 10.4161/auto.5.8.9991 19844159 PMC2873687

[88] Ro SH, Jung CH, Hahn WS, Xu X, Kim YM, Yun YS, Park JM, Kim KH, Seo M, Ha TY, Arriaga EA, Bernlohr DA, Kim DH. Distinct functions of Ulk1 and Ulk2 in the regulation of lipid metabolism in adipocytes. Autophagy. 2013; 9:2103–14. 10.4161/auto.26563 24135897 PMC4028344

[89] Wei R, Cao G, Deng Z, Su J, Cai L. miR-140-5p attenuates chemotherapeutic drug-induced cell death by regulating autophagy through inositol 1,4,5-trisphosphate kinase 2 (IP3k2) in human osteosarcoma cells. Biosci Rep. 2016; 36:e00392. 10.1042/BSR20160238 27582507 PMC5064456

[90] Wang Z, Hu J, Pan Y, Shan Y, Jiang L, Qi X, Jia L. miR-140-5p/miR-149 affects chondrocyte proliferation, apoptosis, and autophagy by targeting FUT1 in osteoarthritis. Inflammation. 2018; 41:959–71. 10.1007/s10753-018-0750-6 29488053

[91] Lu D, Thum T. RNA-based diagnostic and therapeutic strategies for cardiovascular disease. Nat Rev Cardiol. 2019; 16:661–74. 10.1038/s41569-019-0218-x 31186539

[92] Li H, Han S, Sun Q, Yao Y, Li S, Yuan C, Zhang B, Jing B, Wu J, Song Y, Wang H. Long non-coding RNA CDKN2B-AS1 reduces inflammatory response and promotes cholesterol efflux in atherosclerosis by inhibiting ADAM10 expression. Aging (Albany NY). 2019; 11:1695–715. 10.18632/aging.101863 30926762 PMC6461186

[93] Li X, He X, Wang H, Li M, Huang S, Chen G, Jing Y, Wang S, Chen Y, Liao W, Liao Y, Bin J. Loss of AZIN2 splice variant facilitates endogenous cardiac regeneration. Cardiovasc Res. 2018; 114:1642–55. 10.1093/cvr/cvy075 29584819 PMC6148334

[94] Wang K, Liu F, Zhou LY, Long B, Yuan SM, Wang Y, Liu CY, Sun T, Zhang XJ, Li PF. The long noncoding RNA CHRF regulates cardiac hypertrophy by targeting miR-489. Circ Res. 2014; 114:1377–88. 10.1161/CIRCRESAHA.114.302476 24557880

[95] Viereck J, Kumarswamy R, Foinquinos A, Xiao K, Avramopoulos P, Kunz M, Dittrich M, Maetzig T, Zimmer K, Remke J, Just A, Fendrich J, Scherf K, et al. Long noncoding RNA chast promotes cardiac remodeling. Sci Transl Med. 2016; 8:326ra22. 10.1126/scitranslmed.aaf1475 26888430

[96] Yorimitsu T, Nair U, Yang Z, Klionsky DJ. Endoplasmic reticulum stress triggers autophagy. J Biol Chem. 2006; 281:30299–304. 10.1074/jbc.M607007200 16901900 PMC1828866

[97] Sharma M, Pandey R, Saluja D. ROS is the major player in regulating altered autophagy and lifespan in sin-3 mutants of C. Elegans. Autophagy. 2018; 14:1239–55. 10.1080/15548627.2018.1474312 29912629 PMC6103711

[98] Azad MB, Chen Y, Henson ES, Cizeau J, McMillan-Ward E, Israels SJ, Gibson SB. Hypoxia induces autophagic cell death in apoptosis-competent cells through a mechanism involving BNIP3. Autophagy. 2008; 4:195–204. 10.4161/auto.5278 18059169 PMC3164855

[99] Debnath J, Baehrecke EH, Kroemer G. Does autophagy contribute to cell death? Autophagy. 2005; 1:66–74. 10.4161/auto.1.2.1738 16874022

[100] Hassanpour M, Rahbarghazi R, Nouri M, Aghamohammadzadeh N, Safaei N, Ahmadi M. Role of autophagy in atherosclerosis: foe or friend? J Inflamm (Lond). 2019; 16:8. 10.1186/s12950-019-0212-4 31073280 PMC6498679

[101] Liu X, Xiao ZD, Han L, Zhang J, Lee SW, Wang W, Lee H, Zhuang L, Chen J, Lin HK, Wang J, Liang H, Gan B. LncRNA NBR2 engages a metabolic checkpoint by regulating AMPK under energy stress. Nat Cell Biol. 2016; 18:431–42. 10.1038/ncb3328 26999735 PMC4814347

